# COMMUNITY-BASED MUSICAL THEATER AND MEANING-MAKING IN LATER LIFE

**DOI:** 10.1093/geroni/igad104.0911

**Published:** 2023-12-21

**Authors:** Justine McGovern

**Affiliations:** Lehman College, Bronx, New York, United States

## Abstract

This presentation will situate the impact of participating in artistic performances for the first time in later life in relevant scholarship. A growing body of research indicates that older adults benefit from active engagement in the arts, and that this can reduce loneliness, depression and isolation, and improve subjective well-being and quality of life. Benefits include strengthening social connections and a sense of belonging, as well as defining and asserting a sense of self. These outcomes can have a positive impact on physical health as well – older adults engaged in the arts experience lower rates of memory loss and balance issues, heart disease and other health challenges than peers not actively engaged with the arts or passively receiving arts. Participation in performance arts can also improve communication across generations and support processing of loss. Senior centers and community centers with senior members rarely, however, position engagement in performing arts such as musical theater front and center. Programming in visual arts (e.g., painting) and traditional arts (e.g., crochet) tends to take precedence, and this can be detrimental to center members for whom participation in performing arts can be transformative. The presentation will explore meaning-making processes embedded in arts performance and argue for promoting performance opportunities among older adults, as well as suggest ways to replicate the model in other settings.

